# Phosphate Deficiency Negatively Affects Early Steps of the Symbiosis between Common Bean and Rhizobia

**DOI:** 10.3390/genes9100498

**Published:** 2018-10-15

**Authors:** Mariel C. Isidra-Arellano, María del Rocio Reyero-Saavedra, Maria del Socorro Sánchez-Correa, Lise Pingault, Sidharth Sen, Trupti Joshi, Lourdes Girard, Norma A. Castro-Guerrero, David G. Mendoza-Cozatl, Marc Libault, Oswaldo Valdés-López

**Affiliations:** 1 Laboratorio de Genómica Funcional de Leguminosas, Facultad de Estudios Superiores Iztacala, Universidad Nacional Autónoma de Mexico, Tlalnepantla 54090, Estado de Mexico, Mexico; caroo_bff@hotmail.com (M.C.I.-A.); maroresa@yahoo.com.mx (M.d.R.R.-S.); sscaronte@gmail.com (M.d.S.S.-C.); 2 Posgrado en Ciencias Biológicas, Universidad Nacional Autónoma de Mexico, Coyoacan 04510, Ciudad de Mexico, Mexico; 3 Department of Agronomy and Horticulture, University of Nebraska-Lincoln, Beadle Center, Lincoln, NE 68503, USA; lise.pingault@unl.edu (L.P.); marc.libault@unl.edu (M.L.); 4 Informatics Institute, University of Missouri, Columbia, MO 65211, USA; ssz74@mail.missouri.edu (S.S.); joshitr@health.missouri.edu (T.J.); 5 Christopher S. Bond Life Sciences Center, University of Missouri, Columbia, MO 65211, USA; 6 Department of Health Management and Informatics, School of Medicine, University of Missouri, Columbia, MO 65211, USA; 7 Departamento de Biología de Sistemas y Biología Sintética, Centro de Ciencias Genómicas, Universidad Nacional Autónoma de Mexico, Cuernavaca 62210, Morelos, Mexico; girard@ccg.unam.mx; 8 Division of Plant Sciences, C. S. Bond Life Sciences Center, University of Missouri, Columbia, MO 65211, USA; castroguerreron@missouri.edu (N.A.C.-G.); mendozacod@missouri.edu (D.G.M.-C.)

**Keywords:** phosphorus deficiency, common bean, rhizobia, molecular dialog, symbiosis, gene transcription

## Abstract

Phosphate (Pi) deficiency reduces nodule formation and development in different legume species including common bean. Despite significant progress in the understanding of the genetic responses underlying the adaptation of nodules to Pi deficiency, it is still unclear whether this nutritional deficiency interferes with the molecular dialogue between legumes and rhizobia. If so, what part of the molecular dialogue is impaired? In this study, we provide evidence demonstrating that Pi deficiency negatively affects critical early molecular and physiological responses that are required for a successful symbiosis between common bean and rhizobia. We demonstrated that the infection thread formation and the expression of *PvNSP2*, *PvNIN*, and *PvFLOT2*, which are genes controlling the nodulation process were significantly reduced in Pi-deficient common bean seedlings. In addition, whole-genome transcriptional analysis revealed that the expression of hormones-related genes is compromised in Pi-deficient seedlings inoculated with rhizobia. Moreover, we showed that regardless of the presence or absence of rhizobia, the expression of *PvRIC1* and *PvRIC2*, two genes participating in the autoregulation of nodule numbers, was higher in Pi-deficient seedlings compared to control seedlings. The data presented in this study provides a mechanistic model to better understand how Pi deficiency impacts the early steps of the symbiosis between common bean and rhizobia.

## 1. Introduction

*Phaseolus vulgaris* (common bean) represents the principal source of non-animal proteins and minerals for human consumption in the developing world [1]. Additionally, common bean also provides a variety of secondary metabolites with analgesic and neuroprotective properties [2,3]. Similar to other legumes, common bean is able to symbiotically interacts with atmospheric nitrogen-fixing soil bacteria (rhizobia) and incorporates the fixed-nitrogen (e.g., ammonium) into the food chain [1]. Due to its agronomical, medical and ecological properties, common bean is one of the most important legumes worldwide.

To establish a successful symbiosis with rhizobia, a molecular dialogue between legumes and these soil microbes is required. This molecular dialogue begins with the detection of legume-derived flavonoids by compatible rhizobia [4,5]. In response, rhizobia produce diffusible nodulation factors (NF) which are perceived by legumes via the LysM-domain receptor kinase Nod Factors Receptor 5 (NFR5) and NFR1. These receptors are both located at the root hair plasma membrane similarly to the epidermal LysM receptor eNFR [6,7]. Upon NF perception, a wide variety of molecular responses occurs, which is critical to activate the cellular responses required for rhizobial infection and the formation of the new organ termed nodule [8]. For instance, some of these molecular responses are required to modify the polar growth of the root hair leading to the formation of a curled tip [9,10]. In the center of this curl, an infection chamber is formed where rhizobia are entrapped and multiply to form a micro-colony [11]. Immediately, an infection thread is initiated from the infection chamber and elongates through the nodule primordium located in the root cortex [11,12]. Rhizobia are loaded into the infection thread and subsequently released into the nodule primordium cells where they are differentiated into bacteroids. Then, through the activity of the nitrogenase, the bacteroids fix and assimilate the atmospheric dinitrogen within the plant nodule [13].

Root infection and colonization by rhizobia and nodule formation are controlled by two independent genetic programs, which are tightly coordinated by a core of symbiosis-related genes, which belong to the common symbiosis pathway [8,14]. Among these core genes, the leucine-rich repeat (LRR) receptor-like kinase SYMRK along with the plasma-membrane receptor NFR5 and NFR1 are required to detect and decode the NF signal [15,16]. Downstream to NF perception, different ion channels (e.g., the potassium channel Castor/Pollux and the calcium channels belonging to the CNGC15s family) and nucleoporins (e.g., NENA, NUP83 and NUP85) are activated to initiate and maintain rapid oscillations of the nuclear and perinuclear calcium concentrations (i.e., calcium spiking) [17,18,19,20,21]. These fluxes of calcium are further transduced by a calcium and calmodulin-dependent protein kinase (CCaMK), which, in turn, phosphorylates the transcription factor CYCLOPS [22,23]. CYCLOPS is required to activate other critical transcription factors, including the Nodulation Signaling Pathway 2 (NSP2), Nodule Inception (NIN), Nuclear-Factor YA (NF-YA) and NF-YB as well as Ethylene Responsive Element1 (ERN1) [23,24,25]. The coordinated action of these transcription factors is essential to activate the expression of the genes required for the infection of root hairs by rhizobia [8,14]. Among them, *Flotillin2* (*FLOT2*) is crucial to the formation of the infection thread [26]. In parallel, a second genetic program triggering the formation of the nodule meristem in the roots is activated [8,13]. It has been demonstrated that a delicate balance among the phytohormones auxins, cytokinins, and ethylene is required to trigger formation of the nodule meristem [10,27,28]. Genetic analyses have revealed that mutations in the receptors or transporters of these phytohormones affect nodule development. For example, mutations in the cytokinins receptor MtCRE1/LjLHK1 or in the central regulator of the ethylene signaling EIN2 lead to spontaneous nodulation or over-formation of nodules in the legume models *Lotus japonicus* and *Medicago truncatula*, respectively [29,30].

Because symbiotic nitrogen fixation (SNF) is a high-energy demanding process, legumes tightly regulate the number of infections and nodules per plant [31]. This autoregulation of the nodulation (AON) process is locally and systemically activated by the legume. At the systemic level, the plant-derived CLAVATA3/embryo-surrounding region (CLE) peptides are considered as critical regulators of the AON process [31]. In common bean, two AON *CLE peptide-encoding* genes have been identified, *Rhizobia-Induced CLE1* (*PvRIC1*) and *Rhizobia-Induced CLE2* (*PvRIC2*) [32]. *PvRIC1* expression is induced as early as one-day post rhizobia inoculation, whereas *PvRIC2* expression is approximately induced five days upon rhizobia inoculation [32]. PvRIC1 and PvRIC2 peptides are synthesized in the root and translocated to the leaves via the xylem. Upon perception of PvRIC1 and PvRIC2 peptides by a Leucine-rich Repeat Receptor-like Kinase (LRR-RLK) called PvNARK, the expression of different genes participating in the negative regulation of the meristematic activity of the nodule primordium cell is activated [31,32]. It has been widely demonstrated that mutations in the PvNARK/GmNARK/LjHAR1/MtSUNN receptor lead to a hypernodulation phenotype along with a reduction in plant growth [32,33,34,35,36].

Most of the arable soils where common bean is produced have low levels of phosphorus (P), an essential macronutrient required for optimal plant growth and development. Common bean, like other plants, exclusively takes up P from the soil as inorganic phosphorus (i.e., orthophosphate, Pi) [37,38]. However, soil Pi rapidly interacts with Fe^3+^, Ca^2+^, and Al^+3^ ions, forming complexes making Pi unavailable for plant uptake [39]. Pi deficiency constrains common bean growth and productivity as well as its ability to form nodules and symbiotically fix atmospheric nitrogen [40]. It has been reported that Pi deficiency decreases common bean nitrogen-fixation efficiency by increasing the permeability of the nodule to oxygen leading to the inactivation of the rhizobia-biosynthesized nitrogenase [41,42]. Furthermore, it has also been shown that Pi deficiency reduces by more than 60% the number of nodules in common bean plants [40].

Different efforts to understand how legumes cope with Pi deficiency while symbiotically interacting with rhizobia have been made [43,44,45,46]. Physiological studies on different legumes have demonstrated that under Pi deficiency conditions, Pi is preferentially relocated from other organs to the nodules [47,48,49,50,51,52]. Furthermore, both transcriptional and metabolic studies in common bean, *M. truncatula*, and chickpea have revealed a more efficient use of internal Pi sources and the maintenance of Pi homeostasis under limiting conditions [40,43,46]. For instance, it has been reported that the concentrations of different organic acids (e.g., citrate, succinate and pyruvate) were differentially regulated in nodules of both common bean and chickpea plants [40,43,46]. Among them, many are participating in the cation exchange that is required for the Pi uptake. Additionally, it has also been reported that the expression of flavonoid-synthesis related genes decreased in Pi-deficient *M. truncatula* roots [43].

Nodule efficiency and the molecular responses underlying the adaptation of the nodule to Pi deficiency have been studied. However, the molecular mechanisms leading to a reduction in nodule number under Pi deficiency remain unknown. For instance, it is still unclear whether Pi deficiency interferes with the molecular dialogue between common bean and rhizobia. If so, what part of this molecular dialogue is impaired?

In this study, we report the early molecular and physiological responses triggered by rhizobia in common bean seedlings growing under Pi deficient conditions. A transcriptional analysis by quantitative real-time polymerase chain reaction (qRT-PCR) revealed that the expression of *PvNSP2*, *PvNIN* and *PvFLOT2*, was quickly reduced by 50% in Pi-deficient common bean seedlings. Although curled root hairs were observed regardless of the Pi condition (optimal or deficient conditions), their number was severely reduced in Pi-deficient seedlings. Interestingly, the majority of the rhizobia-induced curled root hairs in Pi-deficient seedlings showed aberrant deformations that have previously been associated with defects in the rhizobia infection process. Additionally, we also observed that the expression of the *PvRIC1* and *PvRIC2* genes, regardless of the mock or rhizobia treatment, significantly increased in Pi-deficient seedlings. To gain more insights about the transcriptional modifications leading to an impaired communication between common bean and rhizobia under Pi-deficient conditions, we performed a genome-wide transcriptomics analysis. This analysis revealed that the expression of genes associated with early steps of this symbiosis (e.g., signal transduction and hormone balance) was affected in Pi-deficient plants. The data presented in this study pave the way to better understand the genetic mechanisms underlying the establishment of the symbiosis between rhizobia and common bean under Pi-deficient conditions.

## 2. Material and Methods

### 2.1. Plant Material

Common bean (*P. vulgaris* L. cv Negro Jamapa) seeds were produced under greenhouse conditions in the Facultad de Estudios Superiores Iztacala, Universidad Nacional Autónoma de México (UNAM), at Tlalnepantla, Estado de Mexico, Mexico. Seeds were surfaced sterilized by soaking in 70% ethanol for 1 min, followed by treatment for 10 min with 10% bleach. Seeds were subsequently washed 10 times in sterile water. Sterilized seeds were germinated for three days at 25 °C in Petri dishes containing wet and sterile germination paper (Anchor paper CO, St. Paul, MN, USA). Petri dishes containing common bean seeds were kept under dark conditions at 25 °C.

### 2.2. Bacterial Strains and Culture Conditions

*Rhizobium tropici* CIAT 899 strain was used to inoculate common bean seedlings. *R. tropici* cells were grown for 2 days at 30 °C on PY medium (5 mg/L peptone; 3 mg/L yeast extract) supplemented with 0.7 M CaCl_2_ and 20 μg/mL nalidixic acid (Nal). After two days, *R. tropici* cells were harvested and resuspended in sterile water at O.D_600_ = 0.3. 1 mL of this bacterial suspension was used to individually inoculate common bean seedlings.

*Escherichia coli* DH5α strain carrying the plasmid pFAJ1700::*pLacZ::uidA* was used as a plasmid donor for triparental mating with *R. tropici*. *E. coli* cells were grown in Luria-Bertani (LB) broth supplemented with 10 μg/mL tetracyclin (Tc) overnight at 37 °C.

*E. coli* pRK2013 strain was used as a helper for triparental mating with *R. tropici*. pRK2013 cells were grown in LB broth supplemented with 30 μg/mL kanamycin overnight at 37 °C.

*R. tropici*::*uidA* strain was obtained by triparental mating using the *E. coli* DH5α strain carrying the plasmid pFAJ1700::*placZ*:*uidA*, containing the *uidA* gene under the *lacZ* promoter as a donor, and the pRK2013 as conjugation helper. *R. tropici* derivatives carrying the plasmid were selected in PY medium as Nal and Tc resistant transjugants. *R. tropici*::*uidA* cells were grown in PY medium supplemented with 0.7 M CaCl_2_, 10 μg/mL Tc, and 20 μg/mL Nal for 2 days at 30 °C. After two days, cells were harvested and resuspended in sterile water at OD_600nm_ = 0.3. 1 mL of this bacterial suspension was used to individually inoculate common bean seedlings.

### 2.3. Treatment

Three-day-old common bean seedlings were transferred into a nitrogen-free Fähraeus medium [53] plates supplemented with 1 mM PO_4_ (optimal-Pi conditions) or 5 μM PO_4_ (low-Pi conditions). Three days after transplanting, seedlings were inoculated with autoclaved-water (Mock) or with *R. tropici* CIAT899. Inoculated seedlings were kept under dark conditions at room temperature. At 1, 24 and 48 h post-inoculation, the zone of the roots susceptible to rhizobium infection (hereafter referred as susceptible zone) was isolated, flash frozen in liquid nitrogen, and stored at −80 °C until used for total RNA extraction. The susceptible zone encompassed a region of ~1 cm and is analogous to the distal region of the elongation zone and the entire differentiated zone of the root [54]. Four biological replicates, each one containing susceptible zones from four different seedlings, were independently generated.

### 2.4. Quantification of Soluble Phosphate

Soluble Pi content was determined in roots from common bean seedlings grown for three days in optimal- or low-Pi conditions using the colorimetric assay reported in [55]. Briefly, roots were harvested, rinsed with ultra-pure water, weighed, immediately homogenized in 10 N trichloroacetic acid and fractioned by centrifugation. A soluble fraction was incubated with the staining solution (350 mM FeSO_4_, 16% (NH_4_)_6_Mo_7_O_24_) for 10 min at room temperature. Optical density was determined at 660 nm. For each experimental condition, five replicates, each one containing four roots from different seedlings, were analyzed.

### 2.5. Root Hair Deformation Analysis

Three-day-old common bean seedlings grown for three days under optimal-Pi or low-Pi conditions were inoculated with 1 mL of *R. tropici* suspension (OD_600nm_ = 0.3). Forty-eight hours after inoculation, roots were collected and stained with methylene blue to maximize contrast, and then observed with a bright-field microscope. A total of 20 independent biological replicates were generated, each one including 10 plants.

### 2.6. Quantification of Number of Infection Threads

To quantify the number of infection threads per root, common bean seedlings grown for three days under optimal-Pi or low-Pi conditions were inoculated with 1 mL of *R. tropici*::*uidA* suspension (OD_600nm_ = 0.3). Three days post-inoculation, susceptible zones were collected, rinsed twice with autoclaved ultra-pure water and immersed in uidA staining solution (0.05% 5-bromo-4-chloro-3-indolxyl-β-d-glucuronic acid, 100 mM sodium phosphate buffer (pH7), 0.5 mM potassium ferrocyanide, 0.5 mM potassium ferricyanide, 10 mM Na_2_EDTA and 0.1% Triton X-100) and incubated for one hour at 37 °C. After rinsing in phosphate buffer, the number of infection threads formed at the susceptible zone was quantified under a bright-field microscopy. Additionally, to evaluate the rhizobia adhesion to the common bean roots, the intensity of the blue precipitate (as a product of the activity of the *R. tropici*::*uidA*) formed in the susceptible zone was densitrometrically quantified. To this end, pictures of susceptible zones showing blue precipitate were captured using a SZX10 stereomicroscope (Olympus, Center Valley, PA, USA) equipped with an Olympus UC50 camera (Olympus). The intensity of the blue precipitate in each susceptible zone was quantified as number of pixels by using the software CellSens Standard (Olympus). For these experiments, four biological replicates, each one with 10 roots from different seedlings, were included.

### 2.7. Gene Expression Analysis

To analyze the expression of *PvNSP2*, *PvNIN*, *PvFLOT2*, *PvRIC1* and *PvRIC2* genes, total RNAs were extracted from 0.5 g of rhizobia-inoculated or mock-inoculated susceptible zones from common bean seedlings growing under optimal-Pi or low-Pi conditions using ZR Plant RNA Miniprep kit (Zymo Research, Irvine, CA, USA) according to manufacturer’s instructions. Genomic DNA (gDNA) was removed from purified RNA using DNaseI RNase-free (Thermo Fisher Scientific, Waltham, MA, USA) according to the manufacturer’s instructions. 1 μg of gDNA-free total RNA was used to synthesize cDNA using Thermo Scientific RevertAid Reverse Transcriptase (Thermo Fisher Scientific, USA) according to manufacturer’s instructions. cDNA samples were used to analyze the expression of the aforementioned genes by qRT-PCR in a Step-One qPCR thermocycler (Applied Biosystems, Foster City, CA, USA). The housekeeping gene *PvActin* was used to normalize gene expression levels [56]. The expression level of different genes was calculated according to the equation E = nP_eff_^(−ΔCt)^ where P_eff_ is the primer set efficiency calculated using LinRegPCR [57] and ΔCt is the difference of the cycle threshold (Ct) values of the housekeeping gene and a given gene. qRT-PCR primers were designed by using the online available software Primer 3 v.0.4.0 [58] and using the following parameters: 80–120 pb as product size, 19–23 nt as primer size, 60 °C as primer temperature melting, and 40–60% of primer GC content. To design primers, the sequence from the 3’UTR region of each gene was obtained from the *P. vulgaris* genome Version 2.1 [59,60]. The nucleotide sequences of the qRT-PCR primers used in this study are provided in Table S1. For this experiment, four biological replicates, each one containing roots from four different seedlings, were included.

### 2.8. Preparation of Messenger RNA-Seq Library and High-Throughput Sequencing

Total RNA was isolated from 0.5 g of 48 h rhizobia-inoculated or mock-inoculated roots from common bean plants growing under optimal-Pi or low-Pi conditions, as described earlier in this paper. Stranded messenger RNA-seq (mRNA-seq) libraries were generated from 1.5 μg of gDNA-free total RNA from each experimental condition and prepared using TruSeq RNA Sample Prep kit (Illumina, San Diego, CA, USA) according to the manufacturer’s instructions. Briefly, sample concentration was determined by Qubit flourometer (Invitrogen, Carlsbad, CA, USA) using the Qubit HS RNA assay kit, and the RNA integrity was checked using the Fragment Analyzer automated electrophoresis system (Advanced Analytical, Ankeny, IA, USA). Poly-A containing mRNAs were purified from total RNAs and subsequently fragmented. cDNAs were generated from fragmented RNAs, and the index-containing adapters were ligated to their ends. The amplified cDNAs were purified by the addition of Axyprep Mag PCR Clean-up beads (Axygen, Corning, NY, USA). The quality of each purified RNA-seq library was evaluated using the Fragment Analyzer automated electrophoresis system, and quantified with the Qubit flourometer using the Qubit HS dsDNA assay kit.

In total, 12-stranded mRNA-seq [two Pi conditions (sufficient and deficient), two treatments (mock or rhizobia), and three independent biological replicates] were high-throughput sequenced in 75 bp single-end at the University of Missouri DNA Core Facility by using an Illumina NextSeq500 sequencer (Illumina, USA) according to the manufacturer’s instructions. Image analysis and base calling were performed using the Illumina pipeline (http://www.illumina.com).

### 2.9. Mapping and Processing mRNA-Seq Reads

mRNA-seq analysis was conducted using a custom in-house developed informatics pipeline which first performs a quality check on the raw sequencing FASTQ files using the tool FastQC [61] and trim-galore tool [62] to remove low-quality reads and trim adaptor sequences. We then indexed the *P. vulgaris* Version 2.1 [59,60] reference genome using Bowtie2 short read aligner [63]. The trimmed mRNAseq reads were then aligned, allowing two mismatches, to this index reference genome using TopHat, which also reports splice junction sites [64].

### 2.10. Quantification and Identification of Differentially Regulated Genes

The next step in our pipeline was to quantify the expression level of each transcript/gene by measuring the level of mRNA-seq alignments using the tool Cufflinks [65]. The quantified expression level was represented by Fragments per Kilobase of transcript per Million mapped reads (FPKM) values. The differential expression levels of quantified genes were then calculated using the tool Cuffdiff [65] which takes the observed log fold change of a gene’s expression from control and treatment samples and then reports if the gene is significantly differentially expressed (*q*-values). The thresholds for Cuffdiff were set at 2-fold change and *q*-value ≤ 0.05 before calling genes to be differentially expressed, which are usually annotated with their unique identifiers defined by the gene models annotation and gene coordinates files. Data obtained from this differentially expression analysis was independently validated by qRT-PCR.

### 2.11. Gene Functional Classification

The biological relevance of the differentially regulated genes was assessed by a gene function enrichment analysis using the method Singular Enrichment Analysis (SEA) available in the web-based tool AgriGo [66,67], followed by a summarization and visualization of statistically significant non-redundant gene ontology (GO) terms by using the web-based tool REVIGO [68,69]. Briefly, GO terms enriched in each set of genes were compared to the *P. vulgaris* Version 2.1 gene reference background [59,60]. *p*-values for the GO terms were obtained through Fisher’s exact test, and a *q*-value was computed to produce lists of significant GO terms with an estimated False discovery rate (FDR) of 5%. Enriched GO terms with *q* < 0.05 were further summarized and visualized on REVIGO. Additionally, MapMan gene functional classification was used [70,71]. For MapMan analysis, the common bean mapping file, available at MapMan website [72], was used.

### 2.12. Statistical Analyses

All the statistical analyses were conducted using R software 3.0.1. The specific tests performed are indicated in the legend of the corresponding figure.

## 3. Results and Discussion

### 3.1. Rhizobia-Induced Root Hair Deformation and Infection Thread Formation is Affected in Pi-Deficient Common Bean Seedlings

It has been demonstrated that the reduction in nodule number is one of the negative effects of Pi deficiency in legumes [40]. However, it is unclear whether this nutritional stress interferes with the molecular dialogue between the common bean and rhizobia; therefore, the precise stages of this molecular dialogue that are potentially affected by Pi deficiency remain unknown. To better characterize the effect of the Pi deficiency in the molecular dialogue between the common bean and rhizobia, we first developed a system which allows us to track early molecular and physiological responses to rhizobia under Pi limiting conditions. This system allows the growth of common bean seedlings under optimal- (1 mM) or low- (5 μM) Pi conditions, as well as under sterile and controlled environmental conditions (Figure S1). By using this system, we were able to obtain Pi-deficient common bean seedlings, which led to a 50% reduction in plant Pi contents (Figure S1). Once we confirmed that our experimental system yields Pi-deficient common bean seedlings, we proceeded to assess early responses to rhizobia in common bean seedlings growing under optimal- or low-Pi conditions.

**Figure 1:**
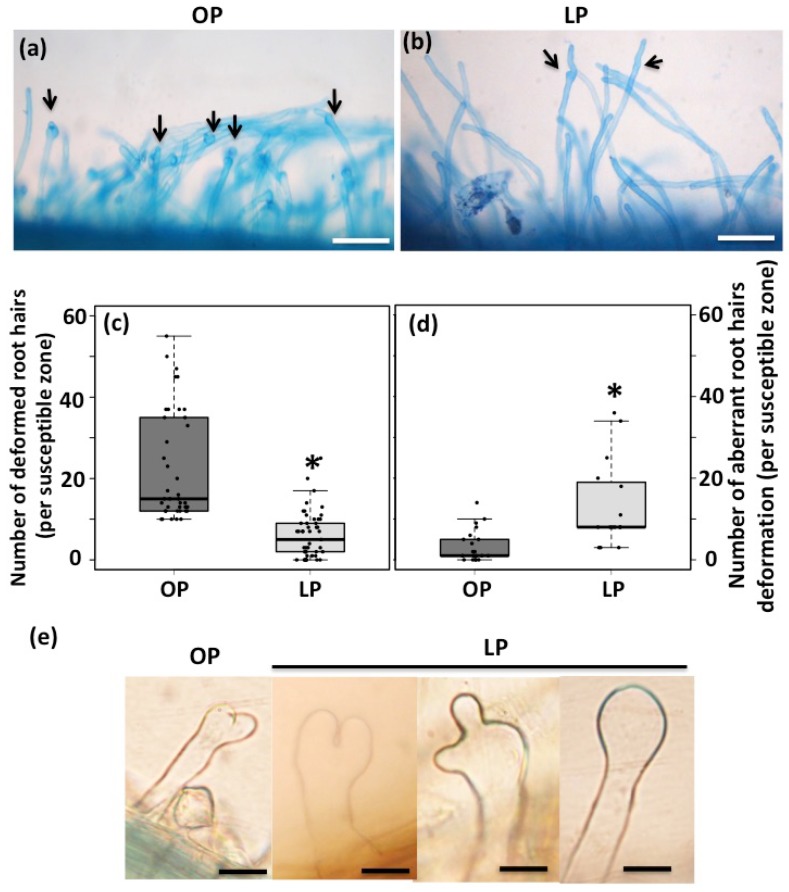
Pi deficiency negatively affects the rhizobia-induced root hairs deformation in common bean. Rhizobia-induced root hairs in common bean seedling growing under optimal-phosphate (Pi) (OP) (**a**) or low-Pi (LP) conditions (**b**). Scale bars in panel represent 100 μm. Arrows indicate some examples of the rhizobia-induced root hairs observed in both OP or LP conditions (**c**) Number of rhizobia-induced root hair deformation in common bean seedlings growing under OP conditions or LP conditions. (**d**) Number of aberrant rhizobia-induced root hair deformations in common bean seedlings growing under OP conditions or LP conditions. Box plots represent first and third quartile (horizontal box side), minimum and maximum (outside whiskers). Asterisk indicates a significant difference according to one-way analysis of variance (ANOVA) (*p*-value < 0.001). Data showed was obtained from 20 biological replicates, each one containing 10 roots from different common bean seedlings. (**e**) Types of aberrant rhizobia-induced root hair deformation observed in common bean seedlings growing under LP conditions. Scale bars in panel represent 50 μm.

**Figure 2:**
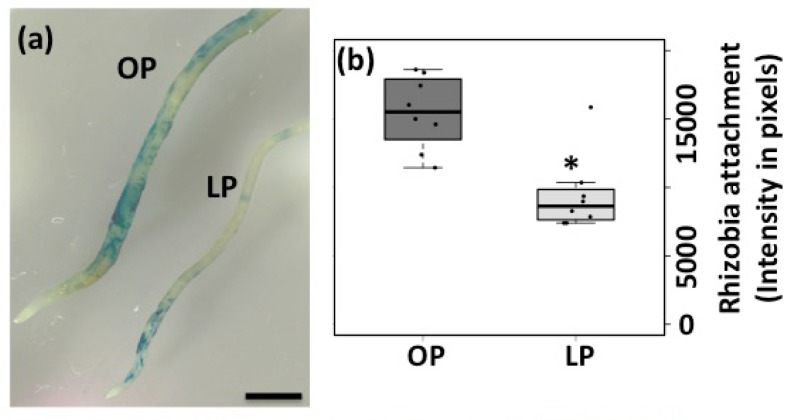

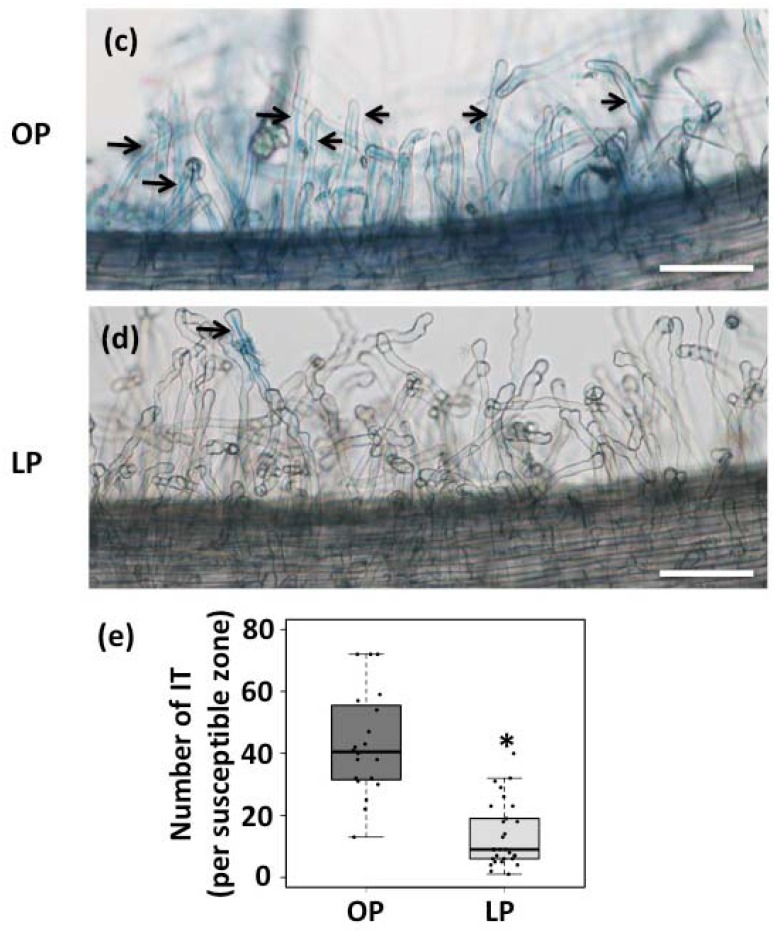
Pi deficiency reduces rhizobia attachment and infection thread formation in common bean. (**a**–**b**) Rhizobia attachment in roots from common bean seedlings growing under optimal-Pi (OP) or low-Pi conditions (LP). Seedlings were inoculated with *R. tropici*::*uidA* and stained by the β-glucorinidase activity. Scale bars in panel represent 2 mm. Infection thread formation in common bean seedlings growing under OP (**c**) or LP conditions (**d**). Arrows indicates some examples of the infection thread observed in OP or LP conditions. Scale bars in panel represent 100 μm. (**e**) Number of infection thread (IT) per susceptible zone. Box plots represent first and third quartile (horizontal box side), minimum and maximum (outside whiskers). Asterisk indicates a significant difference according to one-way ANOVA (*p*-value < 0.001). Data showed was obtained from 20 biological replicates, each one containing ten roots from different common bean seedlings.

One of the early physiological responses triggered by rhizobia is the root hair deformation, which is required for rhizobia entrapping and the formation of the infection chamber [9,10]. To test whether Pi deficiency affects the rhizobia-induced root hair deformation and infection thread formation, common bean seedlings growing for three days under optimal- or low-Pi conditions were inoculated with *R. tropici* CIAT899::*uidA*. Forty-eight hours post inoculation, 95% (n = 190) of the seedlings growing under optimal-Pi conditions showed rhizobia-induced root hair deformation (Figure 1a,c). In contrast, only 50% (n = 200) of the Pi-deficient seedlings displayed deformed root hairs (Figure 1b,c). Interestingly, most of the deformations observed in Pi-deficient common bean seedlings were aberrant (Figure 1d). For instance, we observed root hairs with swelled tip, root hairs with more than one tip outgrowth, and root hairs with spatula-like deformation (Figure 1e). Additionally, we observed that Pi deficiency significantly affected rhizobia attachment, which was reflected by a reduction in the blue precipitate (as a product of the activity of the *R. tropici*::*uidA* strain) at the susceptible zone (Figure 2a,b). Accordingly, we observed that Pi-deficient common bean seedlings developed 60% less infection thread than the seedlings growing under optimal-Pi condition and inoculated with rhizobia (Figure 2c–e). Similar aberrant rhizobia-induced root hair deformations have been observed in *M. truncatula ern1/ern2*, *M. truncatula dmi1* and in *L. japonicus scarn* mutant plants [9,53,73,74]. This defect has been associated with the inhibition in the polar growth of the root hair cells, which affects the rhizobia infection process including the infection thread formation [9,73,74]. Additionally, an early study reported that Pi deficiency affects the rhizobia attachment to Medicago roots [75]. Similarly, our data suggest that Pi deficiency negatively affects the polar elongation of the root hairs, likely leading to inefficient attachment and entrapping of rhizobia.

### 3.2. Pi Deficiency Decreases the Expression of Genes Required for Root Hair Curling and Rhizobia Infection

The coordinated action of different transcription factors is essential for a successful symbiosis with rhizobia. Upon NF perception, the transcription factors NSP2 and NSP1 form a DNA binding complex [24,76]. This complex positively regulates the expression of the transcription factor *NIN*, which is required to activate the expression of different genes involved in rhizobia infection and colonization processes [77]. It has been demonstrated that mutations in the *NSP2* gene significantly affect the rhizobia-induced root hair deformation and rhizobia infection process, particularly in the formation of the infection chamber and infection thread [74,78]. Likewise, early reports have demonstrated that *M. truncatula nin* mutants undergo excessive rhizobia-induced root hair curling but are impaired in infection and nodule formation [77].

**Figure 3:**
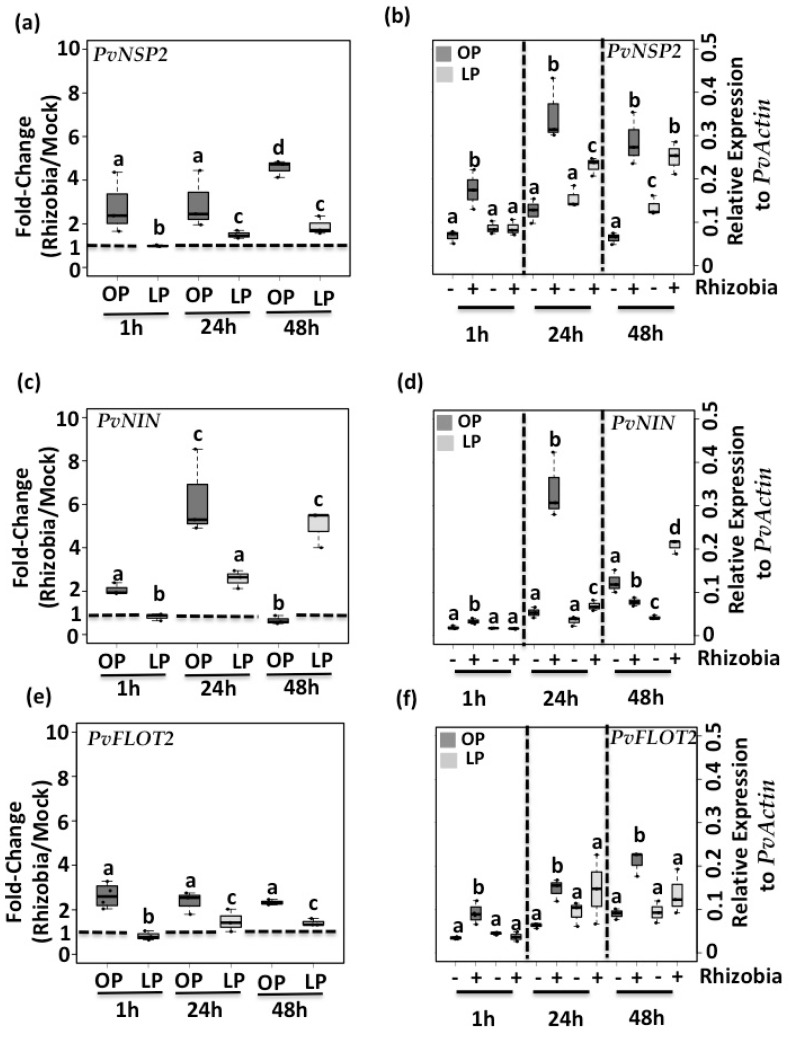
Pi deficiency reduces the activation of early symbiotic genes. Rhizobia-triggered expression of *PvNSP2* (**a**), *PvNIN* (**c**) and *PvFLOT2* (**e**) genes in common bean seedlings growing under OP or LP conditions and inoculated with mock or rhizobia. Data showed are the fold-changes (e.g., OPR/OPM) obtained from four independent replicates, each one containing susceptible zones from four different plants. Dashed line shows basal expression (e.g., no induction triggered by rhizobia). Expression levels of *PvNSP2* (**b**), *PvNIN* (**d**) and *PvFLOT2* (**f**) genes in common bean seedlings grown under OP (dark-gray) or LP (light-gray) conditions and inoculated with mock (−) or rhizobia (+). Data showed was obtained from four independent replicates, each one containing susceptible zones from four different plants. Box plots represent first and third quartile (horizontal box side), minimum and maximum (outside whiskers). One-way ANOVA, followed by a Tukey honest significant difference (HSD) test was performed (*p*-value < 0.01). Statistical classes sharing a letter are not significantly different.

Pi deficiency reduces both rhizobia-induced root hair deformation and infection thread formation in common bean seedlings suggesting that the expression of genes participating in the root hair curling and rhizobia infection is compromised under this nutritional condition. To test this hypothesis, we evaluated the expression of *PvNSP2* (Phvul.009G122700), *PvNIN* (Phvul009G115800), and *PvFLOT2* (Phvul.009G090700) in common bean seedlings growing under optimal- and low-Pi conditions and inoculated with rhizobia. Our analysis revealed that the expression of these three symbiosis-related genes was induced upon one-, 24, and 48 h post-inoculation with *R. tropici* in common bean seedlings growing under optimal Pi conditions (Figure 3). In contrast, we observed a 50% reduction in the expression of these three symbiotic genes in Pi-deficient common bean seedlings inoculated with rhizobia (Figure 3). Interestingly, at 48 h post-inoculation with rhizobia, *PvNIN* was the only gene that showed a substantial induction (in average by 5 fold-change) in Pi-deficient seedlings in response to rhizobia (Figure 3c).

To explore the mechanism behind the reduction in the fold-change expression of these three symbiosis-related genes in response to Pi-deficiency, we analyzed their expression levels in response to mock and rhizobia treatment (Figure 3b,d,f). By comparing the expression levels of *PvNSP2* and *PvNIN* from both optimal-Pi and Pi-deficient seedlings, we did not find significant differences at one and 24 h post-mock treatment (Figure 3b,d). Similarly, we did not find significant differences in the expression levels of *PvFLOT2* across the entire time-course when comparing optimal- and low-Pi seedlings post-mock treatment (Figure 3f). At 48 h, we observed an increase in the expression levels of *PvNSP2* and a diminution in the expression levels of *PvNIN* in mock-inoculated Pi-deficient seedlings. By comparing the expression levels obtained from mock- and rhizobia-inoculated Pi-deficient seedlings, we observed a significant increase in the expression levels of *PvNSP2* and *PvNIN* upon 24 and 48 h of inoculation with rhizobia (Figure 3b,d). Altogether, these transcriptome analyses suggest that the defects in root hair deformation and infection thread formation observed in common bean seedlings grown under Pi-deficient conditions are the consequences of an inefficient activation of critical transcription factors like *PvNIN* and *PvNSP2*, and symbiotic genes like *PvFLOT2*. The fact that substantial induction of the expression of *PvNIN* in Pi-deficient seedlings was detected upon 48 h post-inoculation with rhizobia only also indicates that Pi deficiency delays the activation of critical symbiosis-related genes.

### 3.3. Pi Deficiency Increases the Expression of the PvRIC1 and PvRIC2 Genes in Common Bean Seedlings

**Figure 4:**
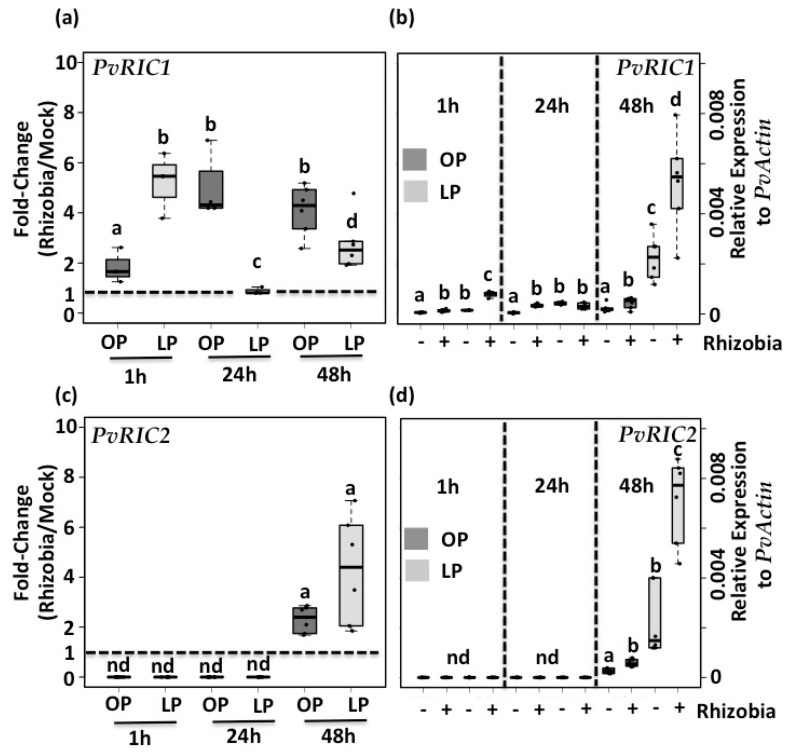
Pi deficiency increases the expression of *PvRIC1* and *PvRIC2* even in the absence of rhizobia. Rhizobia-triggered expression of *PvRIC1* (**a**), and *PvRIC2* (**c**) genes in common bean seedlings growing under OP or LP conditions and inoculated with mock or rhizobia. Data showed are the fold-changes (e.g., OPR/OPM) obtained from four independent replicates, each one containing susceptible zones from four different plants. Dashed line shows basal expression (e.g., no induction triggered by rhizobia). Expression levels of *PvRIC1* (**b**) and *PvRIC2* (**d**) at various times (1, 24 and 48 h) post inoculation with mock (−) or rhizobia (+) in common bean seedlings growing under OP (dark-gray) or LP (light-gray) conditions. Despite several attempts, we were not able to detect reproducible and consistent expression (non-detected: ND) of *PvRIC2* at one and 24 h post-inoculation with mock or rhizobia. Data showed was obtained from four independent replicates, each one containing susceptible zones from four different plants. Box plots represent first and third quartile (horizontal box side), minimum and maximum (outside whiskers). One-way ANOVA, followed by a Tukey HSD test was performed (*p*-value < 0.01). Statistical classes sharing a letter are not significantly different.

It has been reported that the expression of *PvRIC1* and *PvRIC2* is induced one- and five-days post-inoculation with rhizobia, respectively [32]. Based on the fact that Pi deficiency triggers a reduction in the number of nodules in common bean [40] and that the peptides PvRIC1 and PvRIC2 actively participate in the AON process, we hypothesized that the expression of *PvRIC1* and *PvRIC2* might be affected in Pi-deficient common bean seedlings. To test this hypothesis, we quantified the expression of *PvRIC1* (Phvul.005G096901) and *PvRIC2* (Phvul011G135900) in common bean seedlings grown under optimal- or low-Pi conditions, and under mock and rhizobia-treated conditions. This analysis revealed that the expression of *PvRIC1* is induced as early as one-hour post inoculation with rhizobia in both control and Pi-deficient common bean seedlings and have its maximum induction level at 48 h post-inoculation with rhizobia (Figure 4a). In contrast, we were not able to detect a consistent and reproducible induction of *PvRIC2* at 1 and 24 h post-inoculation with rhizobia. However, we observed a clear induction of the expression of *PvRIC2* in response to rhizobia in both control and Pi-deficient common bean seedlings after 48 h post-inoculation (Figure 4c). Although the induction of the expression of these two AON-related genes was observed in both control and Pi-deficient seedlings, we observed that the expression levels of both *PvRIC1* and *PvRIC2* genes were always higher in Pi-deficient seedlings than in control seedlings (Figure 4b,d). Additionally, by comparing the expression levels of mock-inoculated seedlings, we observed that Pi-deficient seedlings always showed higher expression levels similar to those observed in control-seedlings inoculated with rhizobia (Figure 4b,d). Altogether, these data indicate that Pi deficiency induces the expression of *PvRIC1* and *PvRIC2* in the absence of rhizobia, and that the expression of these AON-related genes is highly induced in response to rhizobia in Pi-deficient common bean seedlings. Under this scenario, it is likely that: (1) Pi-deficient common bean seedlings are preconditioned to reduce the number of rhizobia infections. This hypothesis can be supported by the fact that the overexpression of *RIC1* and *RIC2* inhibits nodulation in soybean [79]. (2) Higher expression of *PvRIC1* and *PvRIC2* in response to Pi deficiency might be critical for common bean to properly adapt to Pi deprivation. This hypothesis is supported by the fact that other CLE peptides (e.g., CLE14) [80] play a critical role in the plant adaptation to Pi deficiency [81,82].

### 3.4. mRNA-Seq Analysis

To obtain a better insight into the genetic responses of common bean plants leading to a reduced nodulation under Pi-deficient conditions, we conducted an mRNA-seq analysis. For this genome-wide transcriptional analysis, based on our transcriptional data obtained by qRT-PCR, we selected 48 h post-inoculation to capture more transcriptional responses to rhizobia.

A total of 12 cDNA libraries were generated from control (optimal Pi: OP) and Pi-deficient roots (low Pi: LP) inoculated 48 h with mock (OPM or LPM, respectively) or rhizobia (OPR or LPR, respectively). Three independent biological replicates were generated for each condition. These libraries were sequenced using the Illumina NextSeq500 platform. After filtering low-quality reads, a total of 176,365,724 single-end reads (75 bp) were aligned to the *P. vulgaris* genome reference sequence Version 2.1 [59,60] using Bowtie and Tophat software and allowing two mismatches [64]. Of these, 164,037,947 were uniquely mapped to the common bean genome and were used for further analysis. The full RNA-seq datasets generated from the 12 cDNA libraries used in this study were deposited in the Gene Expression Omnibus [83] under the accession number GSE118968.

### 3.5. Global Transcriptional Responses of Pi-Deficient Common Bean Seedlings Interacting with Rhizobia

In order to identify additional genes with a potential role in the regulation of the early stages of the symbiosis between common bean and rhizobia under low-Pi conditions, the mRNA-seq data sets were analyzed using Cuffdiff [84] with an additional cutoff of 2-fold in pairwise comparisons (e.g., OPR/OPM). In total, 867 (511 up-regulated and 356 down-regulated) and 383 (285 up-regulated and 98 down-regulated) differentially regulated genes were identified when comparing OPR/OPM and LPR/LPM, respectively (Tables S2 and S3). Among them, *PvNSP2* (Phvul.009G122700) and *PvFLOT2* (Phvul.009G090700) were also classified as differentially regulated by mRNA-seq approach (Tables S2 and S3). However, we did not detect the expression of *PvNIN* (Phvul009G115800) in our mRNA-seq analysis, which we explain by a difference in the sensitivity of mRNA-seq and qRT-PCR approaches.

To confirm these mRNA-seq results, the expression of 25 randomly selected genes was analyzed via qRT-PCR and using independent biological material from the one used for mRNA-seq analysis (Figure S2). The expression pattern obtained by qRT-PCR showed the same trend observed by mRNA-seq in response to rhizobia. We found some differences in the fold-change computed by the two independent methods, which we explain by the relative sensitivity and data processing (e.g., algorithms used for data normalization) of each method, along with technical and biological aspects (e.g., efficiency/specificity of qRT-PCR and inherent variation in the responses of common bean to both Pi availability and rhizobia). In spite of these differences, the fold-changes computed for each analyzed gene by qRT-PCR were statistically different between both Pi conditions.

**Figure 5:**
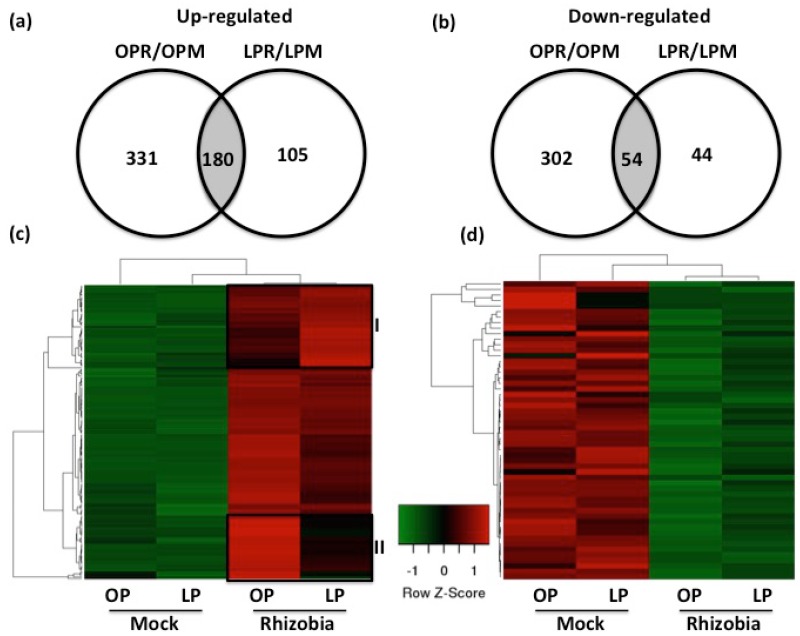
Expression of rhizobia-responsive genes is affected in Pi-deficient common bean seedlings. Number of over-lapping and non-overlapping up-regulated (**a**) or down-regulated (**b**) rhizobia-induced genes among common bean seedlings grown under OP or LP conditions and inoculated with rhizobia (R) or mock (M) during 48 h. Differentially regulated genes in each Pi conditions were identified by Cuffdiff at false discovery rate (FDR) < 0.05, with an additional cutoff of two-fold in a pairwise comparison between treatments (e.g., OPrhizobia/OPmock). Over- and non-overlapping genes were identified after a pairwise comparison between the differentially regulated genes identified in each Pi conditions. A heat map showing the expression levels of up-reguled genes (**c**) or down-regulated genes (**d**) from common bean seedlings growing under OP or LP conditions and inoculated with mock or rhizobia. Expression levels were false colored. Genes showing higher expression levels are shown in different scales of red color, whereas genes showing lower expression levels are showed in different scales of green color.

Upon confirmation of the reliability of our mRNA-seq datasets, we performed additional pairwise comparisons to identify genes that might explain the reduction in the nodule number triggered by Pi deficiency. To this end, we compared the differentially regulated genes found in the pairwise comparisons OPR/OPM and LPR/LPM to identify commonly and uniquely regulated genes under these Pi conditions and interacting with rhizobia. This comparative analysis revealed that 234 (180 up-regulated and 54 down-regulated) genes were commonly regulated in both Pi conditions while interacting with rhizobia (Figure 5a,b and Table S4). In contrast, 633 (331 up-regulated and 302 down-regulated) and 149 (105 up-regulated and 44 down-regulated) genes were uniquely regulated in Pi-optimal and Pi-deficient seedlings, respectively (Figure 5a,b and Tables S5 and S6).

Further hierarchical clustering analyses on the 234 common regulated genes revealed a clear variation in the expression levels computed for these genes (Figure 5c,d). For instance, we identified two clusters showing obvious differences when comparing the expression levels computed for both Pi-optimal and Pi-deficient seedlings inoculated with rhizobia (Figure 5c). The first identified cluster (cluster I) contains genes whose expression levels were higher in rhizobia-inoculated Pi-deficient seedlings than in rhizobia-inoculated optimal-Pi seedlings, whereas the second cluster (cluster II) contains genes whose expression levels were higher in optimal-Pi seedlings than in Pi-deficient seedlings, both of them inoculated with rhizobia (Figure 5c). To extend this analysis and identify genes differentially regulated between Pi-optimal and Pi-deficient seedlings while interacting with rhizobia, we performed a pair-wise comparison of the expression levels between rhizobia-inoculated optimal-Pi and rhizobia-inoculated Pi-deficient conditions. This comparison led us to the identification of 131 differentially expressed genes (77 showing higher expression in Pi-deficient seedlings and 54 showing higher expression in control seedlings) (Figure S3 and Table S7). The observed variation in the expression of these 131 genes was on average five-fold. For instance, Phvul.001G226900 and Phvul.008G244400, which encode for a Wall-associated receptor kinase galacturonan-binding protein and Leucine-Rich Repeat Protein Kinase-Like protein, respectively, showed in average by a four-fold increase in control common bean seedlings. In contrast, the genes Phvul.001G228500 and Phvul.011G074400, which encode for a U-Box domain-containing protein and Fasciclin-Like Arabinogalactan Protein11, respectively, showed on average by a six-fold increase in Pi-deficient common seedlings. Collectively, this transcriptional data clearly indicate that Pi deficiency affects the activity of different rhizobia-regulated genes by either inactivating, decreasing or increasing their expression in common bean.

### 3.6. Expression of Hormone- and Signal-Transduction-Related Genes Is Compromised in Rhizobia Inoculated Pi-Deficient Common Bean Plants

**Figure 6:**
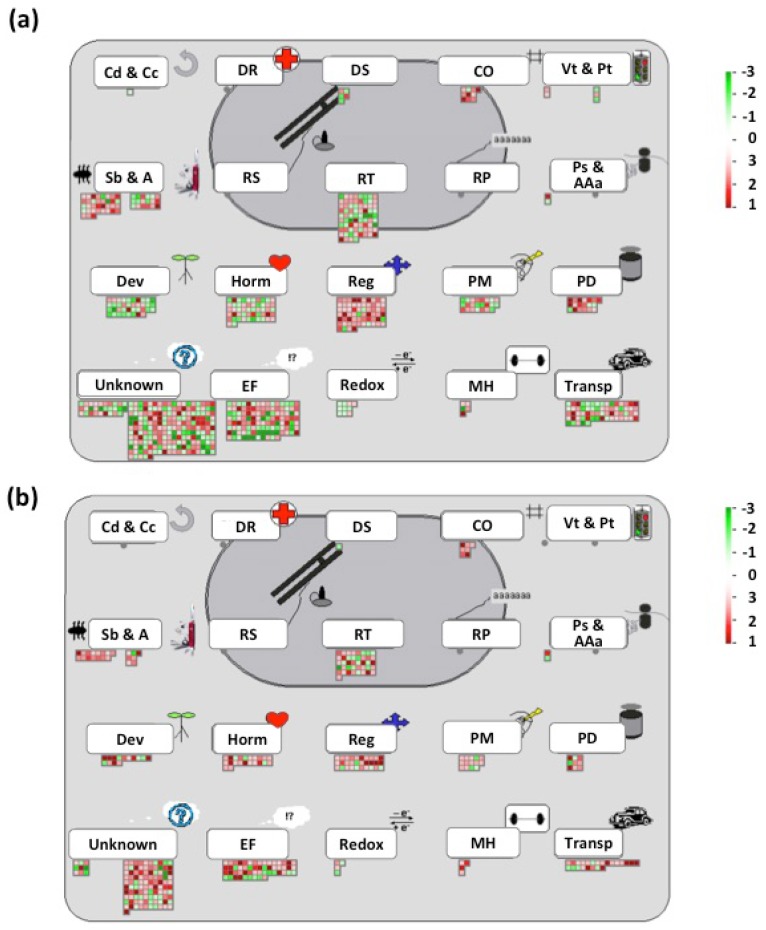
Functional categories affected in Pi-deficient common seedlings inoculated with rhizobia. MapMan regulation overview map showing differences in transcript levels between common bean seedlings growing under OP (**a**) or LP (**b**) conditions and inoculated for 48 h with rhizobia. Fold-changes were false-colored; genes showing higher expression are shown in different scales of red color, whereas genes showing lower expression were colored in green color. The complete sets of genes submitted to MapMan analysis are shown in Tables S2 and S3. Cd and Cc: cell division and cell cycle; DR: DNA repair; DS: DNA synthesis; CO: cell organization; Vt and Pt: vesicle transport and protein targeting; Sb and A: stress biotic and abiotic; RS: RNA synthesis; RT: regulation of transcription; RP: RNA processing; Ps and AAa: protein synthesis and amino acid activation; Dev: development; Horm: hormones; Reg: regulation; PM: protein modification; PD: protein degradation; Unknown: unclassified no ontology and unknown; EF: enzyme families; MH: metal-handling; Transp: transport.

To gain more insights into the molecular functions of the differentially regulated genes identified in this study, we performed a gene-enrichment analysis and a functional classification in MapMan to identify the molecular mechanisms leading to an inefficient symbiosis between common bean and rhizobia under Pi-deficient conditions. This analysis indicated that Regulation of transcription, Hormones, Regulation/Signal transduction, Transport, Biotic interactions and Protein modification/degradation were the most overrepresented functional categories in both control and Pi-deficient common bean seedlings interacting with rhizobia (Figure 6).

Phytohormones play a critical role in different stages of the symbiosis between legumes and rhizobia [85]. For instances, it has been demonstrated that both auxin and cytokinin positively regulate the expression of genes that conforms the common symbiosis pathway, as well as the progression of the symbiotic signaling events from the epidermis to the cortex [27,86]. Recently, it was demonstrated that the auxin accumulation in the root hairs upon rhizobia recognition is essential for the infection thread formation [10]. Furthermore, it has also been demonstrated that the correct perception of cytokinin is crucial for the polar auxin transport and the subsequent auxin accumulation in the rhizobia-infected root hairs [87].

**Figure 7:**
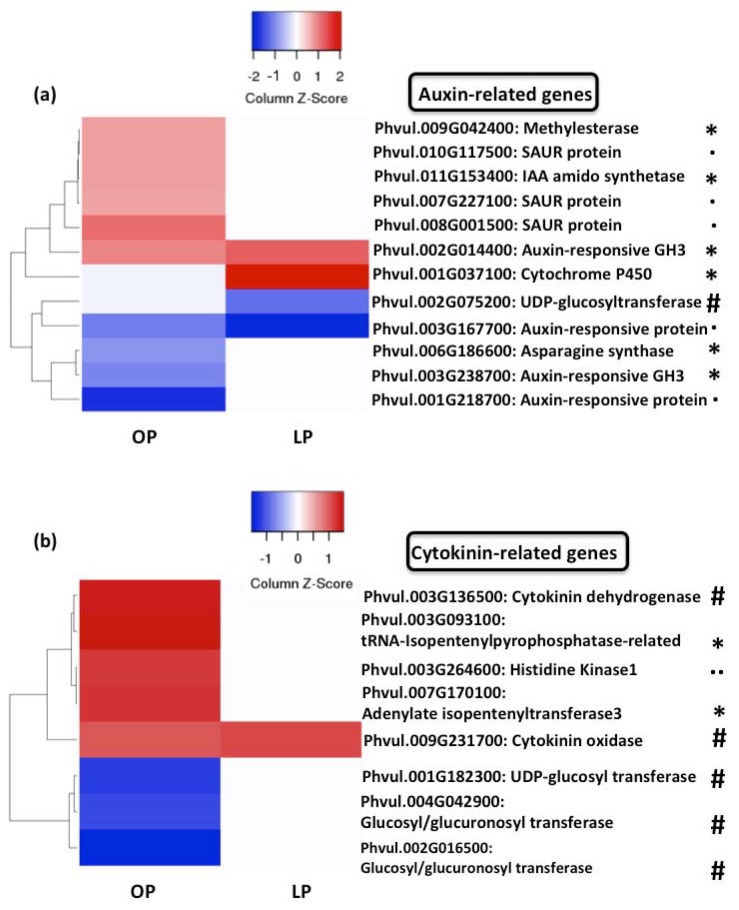
Pi deficiency affects the expression of rhizobia-induced auxin- and cytokinin-related genes. Heat-map showing the fold-change of genes involved in the biosynthesis (*), homeostasis (#), signal transduction (..) of auxin (**a**) or cytokinin (**b**) in common bean seedlings growing under OP or LP conditions and inoculated for 48 h with rhizobia. Genes regulated by auxins or cytokinin are indicated with (.). Fold-changes were false-colored. Genes showing higher expression are shown in different scales of red color, whereas genes showing lower expression were colored in blue color. Genes involved in biosynthesis.

Based on the fact that auxin and cytokinin are crucial regulators of both early and late (e.g., nodule development) stages of the symbiosis between legume and rhizobia [88], and the fact that we found that the Hormones category was one of the overrepresented functional categories, we sought in our transcriptomic data to determinate whether the expression of genes related to these two hormones was affected by Pi deficiency. This analysis revealed that 12 auxin-related genes (six of them involved in auxin biosynthesis, one gene involved in auxin homeostasis and five auxin-responsive genes) and eight cytokinin-related genes (five of them involved in cytokinin homeostasis, two involved in cytokinin biosynthesis, and one involved in cytokinin signaling) were differentially regulated in optimal-Pi common bean seedlings and inoculated with rhizobia (Figure 7a,b). In contrast, only four auxin-related genes (two of them involved in auxin biosynthesis, one involved in auxin homeostasis, and one auxin-responsive gene) and one cytokinin-related (cytokinin homeostasis) gene were differentially regulated in response to rhizobia when common bean seedlings were grown under Pi-deficient condition (Figure 7). The fact that Pi deficiency affects the expression of auxin- and cytokinin-related genes in response to rhizobia, lead us to propose that the defects in early molecular (e.g., expression of *PvNIN* and *PvFLOT2*) and physiological (e.g., root hair deformation) events observed in Pi-deficient common bean seedlings might be partially explained by defects in the auxin-cytokinin balance.

**Figure 8:**
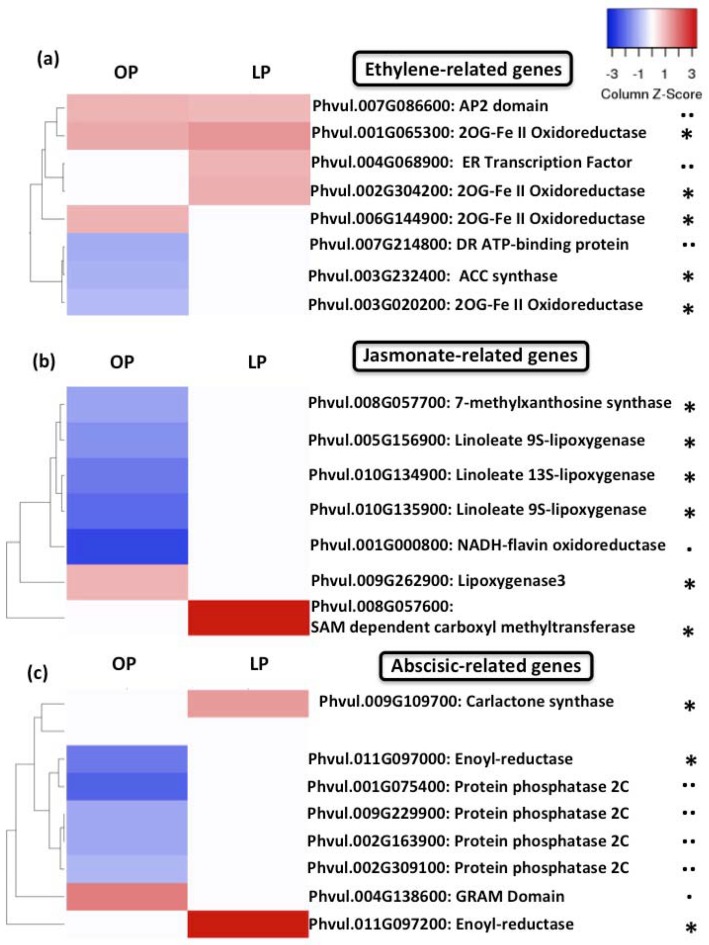
Pi deficiency induce the expression of ethylene-, jasmonate-, and abscisic acid-related genes. Heat-map showing the fold-change of genes involved in the biosynthesis (*), homeostasis (#), signal transduction (..) of ethylene (**a**), jasmonate (**b**) or abscisic acid (**c**) in common bean seedlings growing under OP or LP conditions and inoculated for 48 h with rhizobia. Genes regulated by ethylene, jasmonate or abscisic acid are indicated with (.). Fold-changes were false-colored. Genes showing higher expression are shown in different scales of red color, whereas genes showing lower expression were colored in blue color.

In addition to auxin- and cytokinin-related genes, we also found that genes involved in the biosynthesis of the phytohormones ethylene, abscisic acid (ABA)- and jasmonate, as well as in the signal transduction related to these three phytohormones, were differentially regulated in response to rhizobia inoculation. Although we observed that common bean seedlings growing under optimal-Pi conditions and inoculated with rhizobia showed more differentially regulated genes (six ABA-related genes, seven ethylene-related genes, and six jasmonate-related genes) related to these three hormones than Pi-deficient seedlings inoculated with rhizobia, the majority of them were down-regulated (Figure 8). In contrast, all ethylene-, ABA- and jasmonate-related genes identified in common bean seedlings grown under Pi-deficient conditions and inoculated with rhizobia were up-regulated (Figure 8).

Ethylene, jasmonate, and ABA are essential to activate a series of molecular and physiological responses allowing plants to cope with Pi deficiency [89]. However, growing evidence indicates that these three phytohormones negatively control the establishment of the root nodule symbiosis [90,91,92]. For instances, it has been demonstrated that the accumulation of ethylene or jasmonate inactivates the expression of early symbiosis-related genes (e.g., *ENOD11*), inhibits the initiation and maintenance of the calcium spiking, decreases the number of rhizobia-induced root hair deformation and infection thread, and reduces nodule formation in *M. truncatula* plants [90,91]. Recently, it was demonstrated that the negative impact of ethylene on the rhizobia infection process and nodule formation is under the control of the regulator EIN2 [28]. Hence, our transcriptional data suggest that the activation of genes related to the biosynthesis and signal transduction of these three phytohormones is required in order to allow common bean to properly adapt to Pi deficiency. However, the activation of the ethylene-, jasmonate- and ABA signaling pathways compromise the activation of early molecular and physiological events of the root nodule symbiosis, as we demonstrated in this study.

The proper recognition of rhizobia by the host legume is crucial for a successful symbiosis. To this end, legumes rely on the LysM-domain receptor kinases NFR5 [93], NFR1 [94] and eNFR [7], as well as in the LRR receptor-like kinase SYMRK, to properly detect and decode the NF signal [15,16]. Recently, it was demonstrated that a fourth LysM receptor kinase receptor, EPR3, is required for the perception of compatible rhizobial exopolysaccharides and promotes a successful infection and colonization [95]. Additionally, based on the fact that a gene encoding cell wall-associated receptor kinases (WAKs) is significantly up-regulated upon one-hour treatment with NF in *M. truncatula* roots, it has been proposed that WAKs might play a critical role in the early steps of this symbiosis, primarily in the cell wall-cytoplasm signaling [96]. In our transcriptomic analysis, we also found different types of protein receptors, including LysM receptor kinases, LRR receptor kinases, and WAKs, that were differentially regulated in both control and Pi-deficient seedlings inoculated with rhizobia. However, we observed that Pi-deficient seedlings inoculated with rhizobia showed less differentially regulated genes encoding for protein receptors in response to rhizobia (Figure S4). These data suggest that the misregulation of the expression of additional protein receptor might contribute to an inefficient communication between common bean and rhizobia under Pi-limiting conditions.

## 4. Conclusions

**Figure 9:**
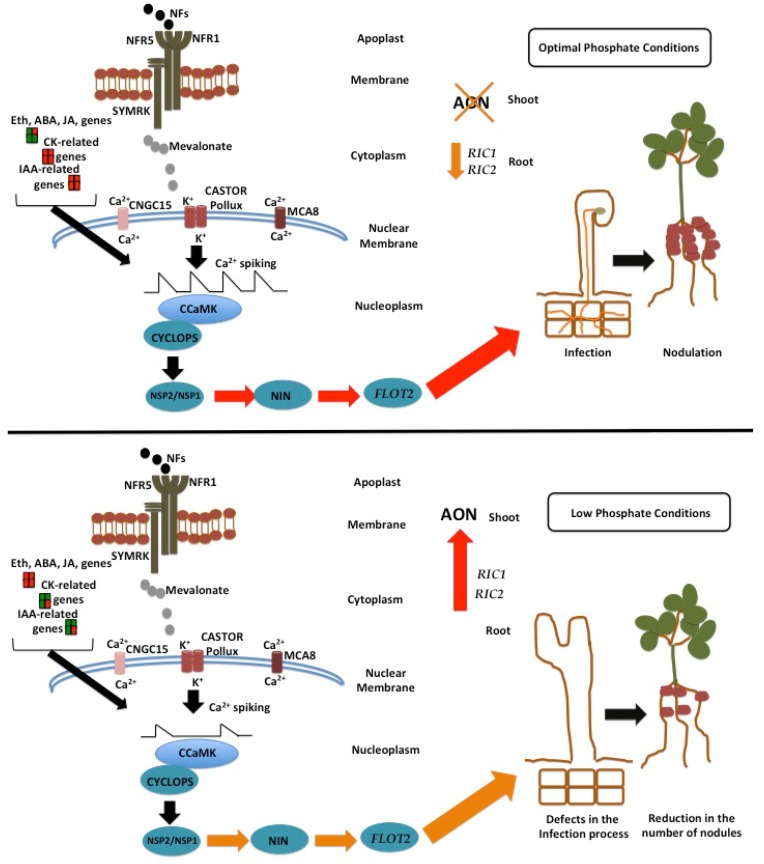
Proposed model of the effects of Pi deficiency in the early steps of the symbiosis between common bean and rhizobia. Higher induction, lower induction and down-regulation of the expression of different rhizobia-induced genes is indicated in red, orange, and green, respectively. Upon NF perception, a variety of molecular responses occurs. For example, the accumulation of mevalonate at the cytoplasm is required for the activation of the Ca^2+^ spiking [97]. These fluxes of calcium are further transduced by CCaMK, which in turn activates the transcription factor CYCLOPS. In turn, CYCLOPS is required to activate the transcription factors NSP2/NSP1 and NIN. The coordinated action of these transcription factors activates the expression of different genes (e.g., *FLOT2*) required for the infection of the root by rhizobia. There is evidence indicating that these molecular events are positively regulated by a delicate balance among the phytohormones auxins (IAA), cytokinins (CK) and ethylene (Eth). These molecular events occur during the first twenty-four hours of interaction between legumes and rhizobia. At this stage, the expression of *RIC1* and *RIC2* genes, which participate in the AON process, is not fully activated. The coordinated action of these molecular events leads to a successful symbiosis. Based on our data, Pi deficiency might affects the phytohormone balance by activating the expression of genes participating in the biosynthesis of abscisic acid (ABA), ethylene (Eth), and jasmonate (JA). These three hormones negatively affect the Ca^2+^ spiking [90,91], leading to a reduction in the expression of symbiosis-related genes (e.g., *NSP2*, *NIN*, and *FLOT2*) required for the rhizobia infection process. Additionally, Pi deficiency induces the expression of *RIC1* and *RIC2*, even in the absence of rhizobia. All these defects in the early stages of this symbiosis lead to a reduction in the number of nodules in common bean.

The data presented in this study lead us to conclude that Pi deficiency negatively affects early events of the symbiosis between legumes and rhizobia. The negative effects in the symbiosis between the common bean and rhizobia under Pi deficiency might be triggered by an imbalance between auxin and cytokinin, as well as by the activation of the ethylene, jasmonate and ABA-related signaling pathway, which are negative regulators of early molecular events of this symbiosis (Figure 9). Finally, the reduction in the number of nodules observed under Pi-deficient conditions can be also explained by a constitutive activation of the AON process (Figure 9); however, further experimentation is needed to confirm this hypothesis.

## Supplementary Materials

The following are available online at http://www.mdpi.com/2073-4425/9/10/498/s1, Figure S1: In vitro system to assess early responses to rhizobia on Pi-deficient common bean seedlings. Seedlings were grown under OP (a) or LP (b) conditions. Seedlings growing under LP conditions show a 50% decrease in their Pi content (c). Asterisk indicates a significant difference according to One-way ANOVA (*p*-value < 0.001), Figure S2: qRT-PCR validation of the mRNA-seq datasets. Data showed are the fold-change obtained from the pairwise comparison between the expression levels obtained from seedlings grown under optimal-Pi (OP) or low-Pi (LP) inoculated with rhizobia (R; OPR or LPR) or mock (M; OPM or LPM), Figure S3: Genes showing significantly higher expression levels in Pi-deficient common seedlings inoculated with rhizobia. Data showed are the expression levels obtained from common bean seedlings grown under optimal-Pi (OP) or low-Pi (LP) conditions and inoculated with rhizobia. Panels on the right site show the functional categories regulated under the described experimental conditions. TF: transcription factors; PM: protein modification; PD: protein degradation; IAA: auxin; ABA: abscisic acid; Eth: ethylene; CK: cytokinins; JA: jasmonate; SA: salicylic acid; GA: gibberellic acid; RK: receptor kinases; CaR: calcium regulation; GP: G-protein; Pi: phosphoinositides; C and Nutr: C and nutrients; MK: MAP kinases; U: unspecified; Thx: thioredoxin; Asc/Glut: arcorbic/gluath; Glutx: glutaredoxin; Px: periredoxin; Dism/Cat: dismutase/catalase, Figure S4: Expression pattern of protein receptors- and secondary metabolism-related genes. Data showed are the fold-change obtained from the pairwise comparison between the expression levels obtained from seedlings grown under optimal-Pi (OP) or low-Pi (LP) inoculated with rhizobia (R; OPR or LPR) or mock (M; OPM or LPM). Fold-changes were false-colored; genes showing higher expression are shown in different scales of red color, whereas genes showing lower expression were colored in green color, Table S1: Primer sequences used for quantitative real-time polymerase chain reaction (qRT-PCR) analysis, Table S2: Rhizobia-induced genes under optimal-Pi conditions, Table S3: Rhizobia-induced genes under low-Pi conditions, Table S4: Commonly regulated genes in both optimal- and low-Pi conditions, Table S5: Genes uniquely regulated under optimal-Pi conditions, Table S6: Genes uniquely regulated under low-Pi conditions, Table S7: Comparison of the expression levels of rhizobia-induced genes under OP or LP conditions.
