# Supplementary figures and images for: Phosphate Deficiency Negatively Affects Early Steps of the Symbiosis between Common Bean and Rhizobia
Source: Genes (Basel). 2018 Oct 15;9(10):498. doi: 10.3390/genes9100498 (PMC6210973; doi:10.3390/genes9100498)

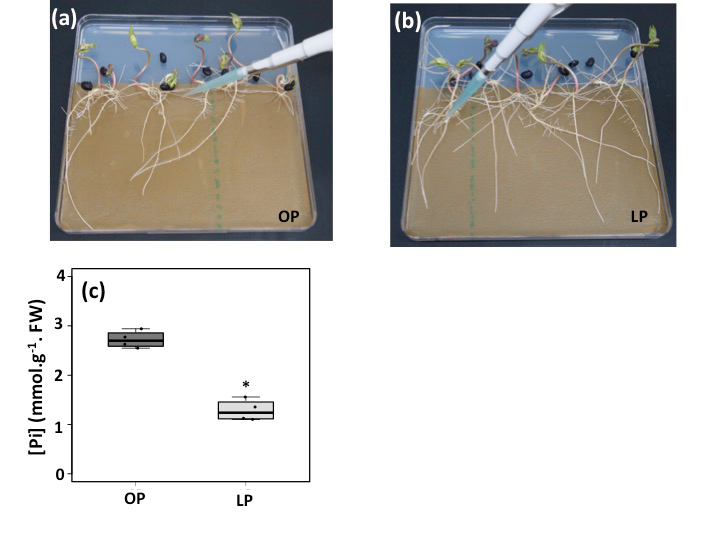

Supplement: Supplementary file 1 [file genes-09-00498-s001.zip › SupplementaryFiles_IAMC/FigS1.tif]

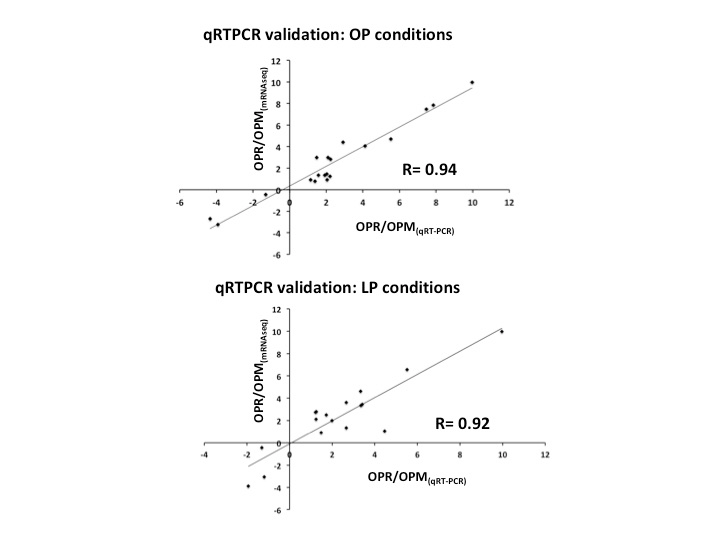

Supplement: Supplementary file 1 [file genes-09-00498-s001.zip › SupplementaryFiles_IAMC/FigS2.tif]

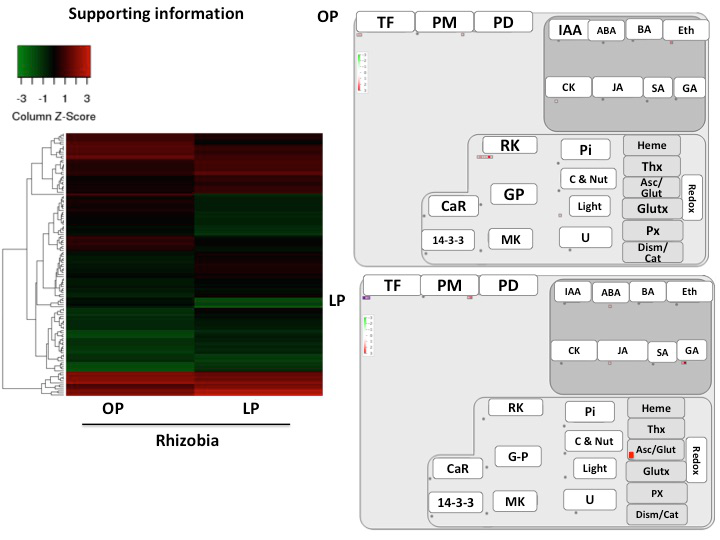

Supplement: Supplementary file 1 [file genes-09-00498-s001.zip › SupplementaryFiles_IAMC/FigS3.tif]

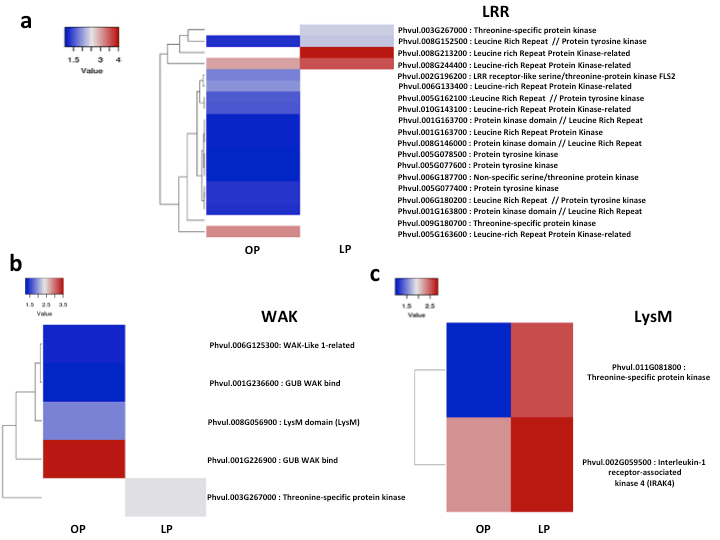

Supplement: Supplementary file 1 [file genes-09-00498-s001.zip › SupplementaryFiles_IAMC/FigS4.tif]
